# Validierung der deutschsprachigen Version des Chronic Ear Survey und dessen psychometrischer Vergleich mit einem etablierten deutschsprachigen Messinstrument

**DOI:** 10.1007/s00106-023-01334-6

**Published:** 2023-08-04

**Authors:** Michael Knoke, Marcus Neudert, Thomas Zahnert, Susen Lailach

**Affiliations:** 1 Klinik für Hals‑, Nasen‑, Ohrenheilkunde, Charité – Universitätsmedizin Berlin, Campus Virchow-Klinikum, Campus Charité Mitte, Augustenburger Platz 1, 13353 Berlin, Deutschland; 2 Klinik und Poliklinik für Hals‑, Nasen- und Ohrenheilkunde, Universitätsklinikum Carl Gustav Carus Dresden, Dresden, Deutschland

**Keywords:** Lebensqualität, Fragebogen, Otitis media, Epidemiologische Messungen, Mittelohr, Quality of life, Questionnaire, Otitis media, Epidemiologic measurements, Middle ear

## Abstract

**Hintergrund:**

Mit dem Chronic Ear Survey (CES) steht seit 2000 ein validiertes Messinstrument zur Erfassung der krankheitsspezifischen bzw. gesundheitsbezogenen Lebensqualität (HRQoL) international zur Verfügung. Ziel der Studie war es, für dieses internationale Messinstrument eine validierte deutschsprachige Fassung zur Verfügung zu stellen und diese mit dem deutschen Chronic Otitis Media Outcome Test 15 (COMOT-15) zu vergleichen.

**Methodik:**

Der CES wurde über einen Vorwärts-rückwärts-Übersetzungsprozess in die deutsche Sprache transferiert. Zur Validierung wurden 79 Patient:innen mit einer COM, bei denen eine Mittelohroperation durchgeführt wurde, prospektiv in die Studie eingeschlossen. Die HRQoL wurde präoperativ und 6 Monate postoperativ mit dem CES und dem COMOT-15 bestimmt. Zu beiden Messzeitpunkten erfolgte auch eine Reintonaudiometrie. In der Kontrolluntersuchung wurde zusätzlich eine rückwirkende Beurteilung der präoperativen Situation anhand des CES und des COMOT-15 zur Bestimmung des Response-Shifts durchgeführt. Als psychometrische Kenndaten wurden die interne Konsistenz, die Test-Retest-Reliabilität, die Diskriminationsvalidität, die Übereinstimmungsvalidität, die Responsivität für beide Messinstrumente bestimmt. Die konvergente Validität beider Messinstrumente wurde anhand einer linearen Regression bewertet.

**Ergebnisse:**

Anhand des CES konnten Patient:innen mit COM von Ohrgesunden sicher unterschieden werden. Der CES zeigte eine sehr gute Reliabilität mit hoher interner Konsistenz (Cronbach‑α 0,65–0,85) und hoher Retest-Reliabilität (r > 0,8). Die globale Einschätzung der Beeinträchtigung der HRQoL korrelierte sehr gut mit den Scores des CES (r = 0,51). Zudem zeigte er eine hohe Änderungssensitivität („standardized response mean“ −0,86). Im Vergleich zum COMOT-15 zeigte sich ein geringerer Response-Shift (Effektstärke −0,17 vs. 0,44). Beide Messinstrumente korrelierten nur gering mit der Luftleitungshörschwelle (r = 0,29 bzw. r = 0,24). Die konkordante Validität beider Messinstrumente war hoch (r = 0,68).

**Schlussfolgerung:**

Die deutsche Version des CES weist zufriedenstellende psychometrische Kenndaten auf, sodass das Einsatz empfohlen werden kann. Der CES legt hierbei den Fokus auf den Einfluss der Ohrsymptomatik auf die HRQoL, wohingegen der COMOT-15 auch funktionelle und psychologische Aspekte miteinschließt. Aufgrund nur geringer Response-Shift-Effekte eignet sich der CES insbesondere für Untersuchungen mit mehreren Wiederholungsmessungen.

## Abstract (en)

**Background:**

With the Chronic Ear Survey (CES), a validated measurement instrument for the assessment of disease-specific health-related quality of life (HRQoL) has been available internationally since 2000. The aim of this study was to provide a validated German version of this international instrument and to compare it with the German Chronic Otitis Media Outcome Test 15 (COMOT-15).

**Methodology:**

The CES was translated into German via a forward-backward translation process. For validation, 79 patients with COM undergoing middle ear surgery were prospectively included. HRQoL was determined preoperatively and 6 months postoperatively using the CES and the COMOT-15. Pure tone audiometry was also performed at both measurement time points. In the control examination, an additional retrospective assessment of the preoperative situation was additionally performed using the CES and the COMOT-15 to assess the response shift. The determined psychometric characteristics were internal consistency, test–retest reliability, discrimination validity, agreement validity, responsiveness, and response shift for both measurement instruments. Convergent validity of both measurement instruments was assessed using linear regression.

**Results:**

On the basis of the CES, patients with COM could be reliably distinguished from patients with healthy ears. The CES showed satisfactory reliability with high internal consistency (Cronbach α 0.65–0.85) and high retest reliability (r > 0.8). The global assessment of HRQoL impairment correlated very well with the scores of the CES (r = 0.51). In addition, it showed a high sensitivity to change (standardized response mean −0.86). Compared to the COMOT-15, it showed a lower response shift (effect size −0.17 vs. 0.44). Both measurement instruments correlated only slightly with air conduction hearing threshold (r = 0.29 and r = 0.24, respectively). The concordant validity of both measurement instruments was high (r = 0.68).

**Conclusion:**

The German version of the CES shows satisfactory psychometric characteristics, so that its use can be recommended. The CES focuses on the influence of ear symptoms on HRQoL, whereas the COMOT-15 also includes functional and psychological aspects. Due to only minor response shift effects, the CES is particularly suitable for studies with multiple repeat measurements.

Die chronische Otitis media (COM) ist eine Erkrankung, welche durch eine Kombination unterschiedlicher Symptome wie Hörverlust, Otorrhö, Otalgie, Kopfschmerzen, aber auch einem Tinnitus gekennzeichnet ist. Vor allem die Einschränkung der Hörfunktion mit einhergehender eingeschränkter Kommunikationsfähigkeit kann zu einem sozialen Rückzug, Niedergeschlagenheit [18] und damit Reduktion der krankheitsspezifischen bzw. gesundheitsbezogenen Lebensqualität („health-related quality of life“, HRQoL) führen [11].

Die HRQoL als Dimension der Ergebnisqualität bei Patient:innen mit COM spielt im deutschsprachigen Raum immer noch eine nachgeordnete Rolle. Die Therapiequalität wird größtenteils weiterhin an audiometrischen Parametern und Einheilungs- bzw. Rezidivraten gemessen. Jedoch zeigte sich in den letzten 15 Jahren eine zunehmende Bedeutung der HRQoL [12].

Initial wurde die HRQoL auch in der Mittelohrchirurgie vorzugsweise mithilfe generischer Messinstrumente, z. B. dem Short Form 36 (SF-36) erfasst, wobei sich anhand der generischen HRQoL-Messung kein Therapieeffekt nachweisen ließ, da in diesen Messinstrumenten die Symptomatik der COM und deren Auswirkungen nur ungenügend abgebildet ist [2, 13, 16]. Dementsprechend sind die Bestrebungen der otologischen Community, zunehmend auch krankheitsspezifische Messinstrumente für die COM zur Verfügung zu stellen, nachvollziehbar und prinzipiell zu begrüßen. Mittlerweile existieren international 6 Messinstrumente zur Erfassung der HRQoL bei Patient:innen mit COM, welche in unterschiedlichen Sprachen validiert vorliegen und sich in ihren erfassten Dimensionen unterscheiden [12]. Nadol et al. stellten 2000 das erste krankheitsspezifische HRQoL-Messinstrumenten für Patient:innen mit COM in englischer Sprache zur Verfügung, den Chronic Ear Survey (CES) [16, 25]. Mittlerweile liegt für dieses Messinstrument auch eine italienische, niederländische und chinesische validierte Fassung vor [7, 21, 25]. Im deutschsprachigen Raum werden mit dem Zurich Middle Ear Inventory 21 (ZCMEI-21) und dem Chronic Otitis Media Outcome Test 15 (COMOT-15) 2 Messinstrumente mit abweichenden Schwerpunkten verwendet [1, 2]. Aufgrund der damit bestehenden nationalen und internationalen Heterogenität von HRQoL-Messinstrumenten ist eine vergleichende Auswertung von HRQoL-Studien kaum möglich, zumal es bislang keine internationalen und nationalen Empfehlungen zur Auswahl eines Messinstruments gibt [8]. Nicht zuletzt wird die Situation durch die Bereitstellung eigener Übersetzungsversionen vorhandener Messinstrumente ohne entsprechende Validierung des Messinstruments erschwert. Eine Translation eines Messinstruments bedarf mehr als einer einfachen Übersetzung. Für jede Übersetzung ist ein Validierungsprozess notwendig, der untersucht, ob das Messinstrument auch das misst, was es vorgibt zu messen.

Um die Heterogenität der Messinstrumente zu bewerten, erfolgte unlängst eine vergleichende Untersuchung zur Bewertung des COMOT-15 und ZCMEI-21 in deutscher Sprache. Beide Messinstrumente wiesen eine hohe Übereinstimmungsvalidität auf, sodass eine Umrechnungsformel zur Schätzung der jeweiligen Gesamtscores beider Messinstrumente zur Verfügung gestellt werden konnte [14].

Ziel der Studie war es daher, zunächst eine deutschsprachige validierte Version des international verbreiteten CES zur Verfügung zu stellen. Darüber hinaus erfolgte ein Abgleich seiner psychometrischen Kenndaten mit dem im deutschen Sprachraum etablierten COMOT-15, um Empfehlungen zur zielorientierenden Anwendung von Patient-Reported Outcome Measures (PROM) im klinischen Alltag und in klinischen Studien abzuleiten.

## Patient:innen und Methoden

Die Studie wurde durch die Ethikkommission des Universitätsklinikums Dresden genehmigt (EK 268072014).

Insgesamt wurden 79 Patient:innen, die sich im Zeitraum von Mai 2016 bis Mai 2018 bei COM in der Universitätsklinik Dresden operieren ließen, prospektiv für die Studie rekrutiert. Ausgeschlossen wurden Patient:innen, die das 18. Lebensjahr nicht vollendet hatten und nicht geschäftsfähig waren, sowie Patient:innen, die sich im Untersuchungszeitraum einer weiteren Ohroperation unterziehen mussten.

Alle Patient:innen erhielten ein Reintonaudiogramm präoperativ sowie zur Kontrolle 6 Monate postoperativ. Zur Auswertung der audiologischen Daten wurde der 4‑Frequenz-Pure-Tone-Average-Wert der Luftleitungsschwelle und der Schallleitungskomponente (Air-Bone Gap, ABG) über die Frequenzen 0,5; 1; 2 und 4 kHz gemittelt. Zu beiden Zeitpunkten wurde die HRQoL anhand der deutschsprachigen Version des CES sowie des bereits validiert in deutscher Sprache vorliegenden COMOT-15-Fragebogens erhoben. Eine weitere Erfassung der Lebensqualität mittels CES und COMOT-15 erfolgte eine Woche nach der Kontrollvorstellung.

Als Kontrollgruppe wurden ohrgesunde Proband:innen mit subjektiver Normalhörigkeit nach Aufklärung über das Studienvorhaben und den Studienablauf einbezogen.

### Chronic Ear Survey

Der CES ist ein psychometrisches Messinstrument, welches anhand von 13 Items die Häufigkeit, Dauer und Schwere der mit einer COM verbundenen Symptome und damit die krankheitsspezifische HRQoL bewertet [16]. Die Antworten jeder Frage variieren von Häufigkeitseinstufungen bis hin zu Likert-skalierten 4‑ bis 6‑stufigen Antwortmöglichkeiten. Dabei ist das Messinstrument in 3 Subscores „Einschränkungen in Aktivitäten“ (Fragen a1–a3), „Symptome“ (Fragen s1–s7) und „Nutzung medizinischer Ressourcen“ (Fragen m1–m3) unterteilt. Jede Frage wird auf eine Skala von 0 bis 100 transformiert. Zur Berechnung des Gesamtscores müssen die Gesamtwerte der Subscores durch die Anzahl der enthaltenen Fragen geteilt werden. Anschließend werden die Subscores addiert und durch 3 geteilt, um den Gesamtwert des CES zu erhalten. Höhere Werte gehen dabei mit einer besseren Lebensqualität einher.

### Chronic Otitis Media Outcome Test 15

Der COMOT-15 wurde als krankheitsspezifisches Messinstrument zur Erfassung der HRQoL bei Patient:innen mit COM primär in deutscher Sprache entwickelt und validiert [2]. Anhand von 15 Items, welche 5‑stufig Likert-skaliert sind, werden neben einem Gesamtscore (Items 1–13) 3 Subscores gebildet: „Ohrsymptome“ (Punkte 1–6), „Hörfunktion“ (Punkte 7–9) und „psychische Gesundheit“ (Items 10–13). Zusätzlich erfolgt eine allgemeine Bewertung der krankheitsspezifischen HRQoL (Item 14) und die Angabe der Häufigkeit von Arztbesuchen als „medizinische Ressourcennutzung“ (Item 15). Zur Bewertung des COMOT-15 wird dieser auf eine Skala von 0 bis 100 transformiert, wobei der Wert 0 die geringste Einschränkung der HRQoL darstellt.

### Validierung des deutschsprachigen CES

Die Nutzung der englischsprachigen Originalversion des Fragebogens wurde durch den Autor genehmigt. Zunächst wurde durch 2 muttersprachliche professionelle Übersetzer die Übersetzung des CES aus dem Englischen ins Deutsche durchgeführt. Die Übereinstimmung zwischen diesen Übersetzungen wurde geprüft, und es wurden keine Abweichungen vom Inhalt des Fragebogens festgestellt. Anschließend übersetzten 2 muttersprachliche Übersetzer den Fragebogen zurück ins Englische. Beide Versionen des Fragebogens wurden durch 2 Otologen überprüft, um zu bestätigen, dass die Fragen verständlich waren und die ursprüngliche Bedeutung erhalten blieb.

**Figure 1:**
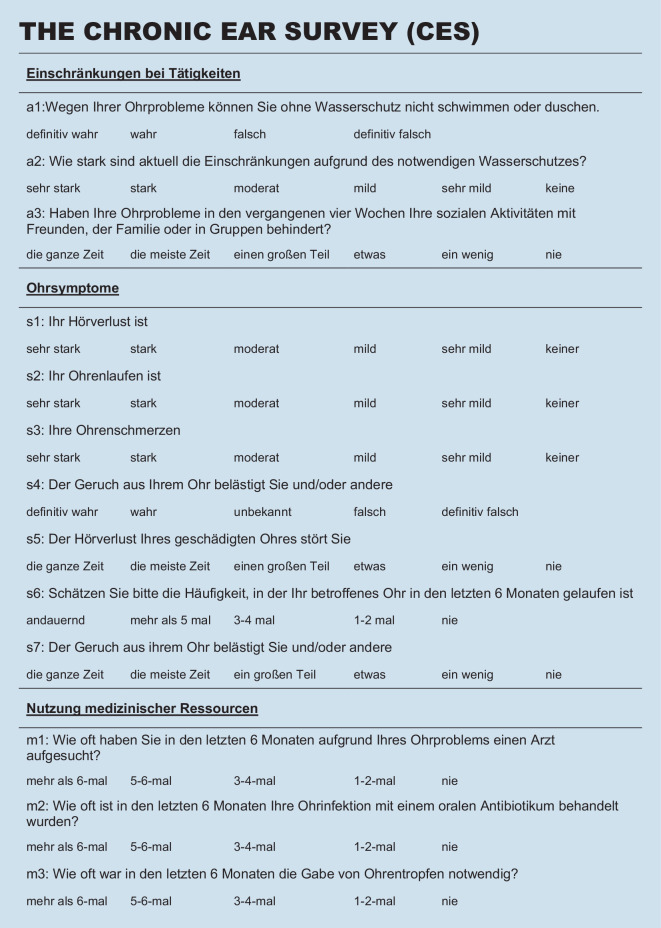

Zur Validierung der deutschen Version des CES (Abb. 1) wurden die Reliabilität, die Validität und die Responsivität des Messinstruments überprüft.

### Response-Shift

Zusätzlich wurde ein möglicher Response-Shift untersucht. Hierunter wird eine Veränderung des Bewertungshintergrunds der HRQoL aufgrund eines kritischen Ereignisses bezeichnet [22]. Ein Response-Shift tritt v. a. auf, wenn sich Menschen mit kritischen und bedrohlichen Lebensereignissen auseinandersetzen [23]. Solche Ereignisse findet man nicht nur in der Onkologie, auch die Mitteilung von Diagnosen oder der Beginn einer Behandlung im Rahmen einer chronischen Erkrankung kann dazu zählen.

Berücksichtigt werden muss ein Response-Shift im Rahmen von Longitudinalmessungen. Je größer ein solcher Response-Shift zwischen 2 Messpunkten ausfällt, desto geringer sind die Differenzwerte der Longitudinalmessung als wahre, quantitative Veränderung der Lebensqualität zu interpretieren. Dabei sollten aber auch Verzerrungen durch fehlerhafte Erinnerung im Rahmen von Then-Testungen berücksichtigt werden [19]. Zur Bestimmung des Response-Shifts erfolgte im Rahmen der Kontrollvorstellung eine zusätzliche rückblickende Einschätzung der HRQoL durch die Patient:innen anhand des CES und des COMOT-15.

### Statistische Auswertung

Zur statistischen Auswertung wurde PASW Statistics 25.0 (Fa. SPSS Inc., Chicago, USA) verwendet. Die Verteilungseigenschaften wurden mittels arithmetischen Mittelwerts und Standardabweichung beschrieben.

Im Rahmen der *Einzelitemanalyse* des CES wurden Mittelwert, Spannweite, Varianz und die Trennschärfe der jeweiligen Items bestimmt. Die Itemschwierigkeit wurde anhand eines Schwierigkeitsindexes durch Bildung des Quotienten aus Itemmittelwert und maximal erreichbarem Wert in einem Item multipliziert mit 100 erfasst. Werte von 15 bis 85 werden als Itemschwierigkeit empfohlen [6]. Items mit einer Itemschwierigkeit < 15 gelten als zu schwer, mit einer Schwierigkeit > 85 als zu leicht. Trennschärfen ab 0,3 wurden als akzeptabel gewertet [15].

Zur Bewertung der *Reliabilität *wurde zunächst die interne Konsistenz des Messinstruments durch das Cronbach‑α für den Gesamt- und die Subscores getestet. Psychometrische Instrumente sollten nur verwendet werden, wenn Cronbach‑α einen Wert ≥ 0,65 erreicht [24]. Außerdem wurde die Test-Retest-Reliabilität überprüft. Alle Patient:innen wurden daher gebeten, postoperativ den Fragebogen 2‑mal zu beantworten: zum Zeitpunkt der Kontrollvorstellung und eine Woche nach der Kontrollvorstellung. Die Ergebnisse des CES zu diesen beiden Messzeitpunkten wurden mithilfe einer Korrelationsanalyse (nach Pearson) bewertet. Dabei gelten Korrelationskoeffizienten von 0,70–0,95 als hoch bis sehr hoch [20].

Zur Bestimmung der *Validität *des Messinstruments wurde zum einen die Übereinstimmungsvalidität durch Bestimmung des Korrelationskoeffizienten nach Pearson für den Gesamt- und die Subscores mit einer globalen krankheitsspezifischen Frage (Frage 14: Gesamteinschätzung der Beeinträchtigung der Lebensqualität durch die Ohrerkrankung) aus dem bereits validiert vorliegenden COMOT-15-Fragebogen erfasst. Zum anderen wurde die Diskriminationsvalidität durch den Vergleich mit einer „ohrgesunden“ Kontrollgruppe (Patient:innen ohne COM) mittels t‑Test ermittelt.

Die *Änderungssensitivität *wurde durch das „standardized response mean“ (SRM) beschrieben, welches als Quotient aus mittlerem Veränderungsscore und der Standardabweichung des Veränderungsscores definiert ist. Werte ≥ 0,8 werden dabei als großer Effekt; ≥ 0,5, < 0,8 als mittlerer Effekt; ≥ 0,2, < 0,5 als kleiner Effekt und < 0,2 als geringfügiger Effekt bewertet [4].

Die Überprüfung eines möglichen *Response-Shifts* erfolgte mittels gepaartem t‑Test im Sinne einer indirekten Veränderungsmessung. Hierbei wird zum zweiten Messzeitpunkt eine rückblickende Einschätzung zum ersten Messzeitpunkt erhoben (Then-Testung). Zur Einschätzung der Größe des Effekts wird die „standardized effect size“ (SES) berechnet. In der Effektstärkeberechnung ist grundlegend, dass die Mittelwertdifferenz aus den 2 Messzeitpunkten an einem Streuungsmaß relativiert wird [9]. Bei der SES wird hierzu die Standardabweichung der Messung präoperativ verwendet. Zur Bewertung von Effektstärken hat sich die Einteilung nach Cohen etabliert [4]. Effekte um 0,2 werden als klein, um 0,5 als mittel und um 0,8 als groß beschrieben. Zusätzlich werden Effektstärken ≥ 0,5 als klinisch relevant angesehen [5, 19].

Die Beziehung zwischen den Gesamtscores des CES und des COMOT-15 wurde anhand einer linearen Regression modelliert. Das Signifikanzniveau wurde mit *p* < 0,05 definiert.

## Ergebnisse

### Patient:innencharakteristik

In die Studie wurden 79 Patient:innen mit einer COM eingeschlossen. Die demografischen Parameter dieser Patient:innengruppe sind Tab. 1 zu entnehmen.

**Table 1:** 

		Patient:innen präoperativ (*n* = 79)
Alter		48,6 ± 14,9 (SD) Jahren
Geschlecht	Weiblich	50 (63 %)
	Männlich	29 (37 %)
Pathologie	Chronische mesotympanale Otitis media	41 (52 %)
	Cholesteatom	38 (48 %)
Hörfunktion	Luftleitung (0,5–4 kHz)	48,09 ± 20,05 dB
	ABG (0,5–4 kHz)	24,25 ± 14,49 dB

*ABG* „air-bone gap“; *SD* „standard deviation“, Standardabweichung

Zur Kontrolluntersuchung 6 Monate postoperativ wurden Daten von 59 Patient:innen erhoben (Antwortrate 75 %). Bei 30 dieser Patient:innen lag ein Cholesteatom und bei 29 eine chronische mesotympanale Otitis media vor.

Die ohrgesunde Kontrollgruppe bestand aus 30 Proband:innen mit einem Durchschnittsalter von 30,9 ± 8,1 Jahren. Die Geschlechtsverteilung war mit 18 Frauen (60 %) und 12 Männern (40 %) vergleichbar mit der Patient:innengruppe (*p* = 0,82). Das Durchschnittsalter war jünger als in der Gruppe mit COM (30,9 ± 8,1 vs. 48,6 ± 14,9 Jahre, *p* < 0,001).

### Psychometrische Kenndaten des deutschsprachigen CES

Der CES wies im Gesamt- und in den Subscores in der deutschsprachigen Version prä- und postoperativ eine akzeptable interne Konsistenz auf (Tab. 2).

**Table 2:** 

	Cronbach‑α präoperativ		Cronbach‑α postoperativ		Test-Retest-Reliabilität (r)		Übereinstimmungsvalidität (r)		SRM		Korrelation (r) mit der Luftleitungsschwelle (0,5–4 kHz)		Response-Shift (SES)
	Eigene Daten	Original	Eigene Daten	Original	Eigene Daten	Original	Eigene Daten	Original	Eigene Daten	Original	Eigene Daten	Original	Eigene Daten
*CES*													
Gesamtscore	0,85	0,83	0,83	–	0,89***	–	0,51***	–	−0,86	0,42	0,29*	0,15	−0,17
Einschränkungen in Aktivitäten	0,65	0,62	0,68	–	0,84***	–	0,41***	–	−0,50	–	0,20	−0,11	−0,21
Symptome	0,84	0,80	0,76	–	0,82***	–	0,51***	–	−0,71	–	0,33**	0,33*	−0,07
Medizinische Ressourcennutzung	0,77	0,75	0,77	–	0,92***	–	0,13	–	−0,64	–	0,10	0,18	−0,19
*COMOT-15*													
Gesamtscore	0,88	0,89	0,88	0,89	0,90***	0,89	0,78***	0,72***	0,79	0,44	0,24	–	0,44
Ohrsymptomatik	0,79	0,91	0,79	0,91	0,85***	0,83	0,59***	0,56***	0,69	0,46	0,08	–	−0,20
Hörfunktion	0,90	0,91	0,91	0,90	0,89***	0,81	0,64***	0,56***	0,46	0,24	0,34**	–	0,59
Psychisches Befinden	0,87	0,90	0,88	0,90	0,82***	0,86	0,73***	0,69***	0,70	0,32	0,19	–	0,37

Die psychometrischen Kenndaten der Originalarbeiten entstammen für den COMOT-15 der Publikation von Baumann et al. 2009 [2] und für den CES der Publikation von Wang et al. 2000 [25]

*–* keine Daten vorhanden, *SES* „standardized effect size“, Effektstärke; *SRM* „standardized response mean“, *CES* Chronic Ear Survey, *COMOT-15* Chronic Otitis Media Outcome Test 15

*p ≤ 0,05 signifikant, **p < 0,01 sehr signifikant, ***p ≤ 0,001 hoch signifikant

Bei der Bestimmung der Test-Retest-Reliabilität ergaben sich Korrelationskoeffizienten von > 0,80 für den Gesamtscore sowie alle Subscores, was eine hohe Test-Retest-Reliabilität impliziert (Tab. 2).

Die Übereinstimmungsvalidität wurde durch Korrelation der globalen gesundheitsspezifischen Frage (Frage 14: Gesamteinschätzung der Beeinträchtigung der Lebensqualität durch die Ohrerkrankung) des COMOT-15 mit den Gesamt- und Subscores des CES getestet. Hierbei zeigte sich eine positive Korrelation sowohl für den Gesamtscore als auch die Subscores „Einschränkung in Aktivitäten“ und „Symptome“ (Tab. 2). Der Subscore „medizinische Ressourcennutzung“ zeigte keine signifikante Korrelation.

**Figure 2:**
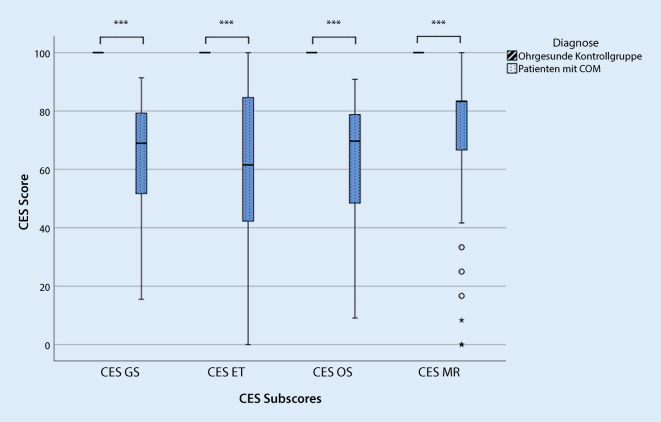

Zur Untersuchung der Diskriminationsfähigkeit wurde die deutsche Übersetzung des CES auch von einer „ohrgesunden“ Kontrollgruppe (*n* = 30) ausgefüllt. In allen Skalen gab die Kontrollgruppe eine signifikant geringere Beeinträchtigung der HRQoL an als die Patient:innengruppe mit COM (Abb. 2).

### Vergleich des deutschsprachigen CES mit dem COMOT-15

**Figure 3:**
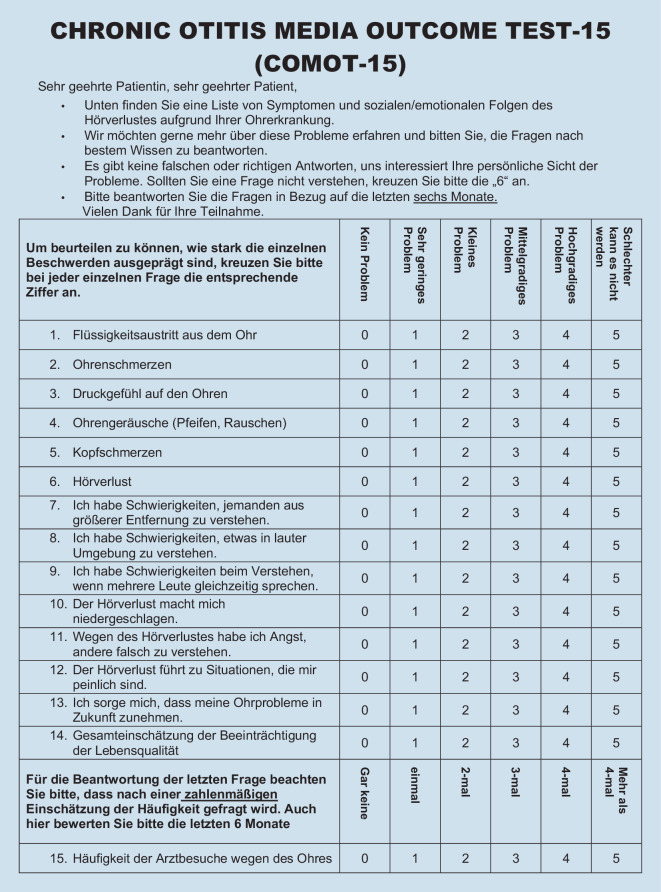

Bei der Bewertung der Itemschwierigkeit (Tab. 3) der einzelnen Items des CES zeigte sich, dass diese mit Ausnahme des Items m3 (Häufigkeit einer oralen Antibiotikagabe, Itemschwierigkeit 85,75) innerhalb der empfohlenen Grenzwerte lagen. Beim COMOT-15 (Abb. 3) lag die Itemschwierigkeit aller Items im empfohlenen Grenzbereich. Während der CES vorwiegend Fragen mittlerer bis leichter Schwierigkeit umfasste, wiesen die Items des COMOT-15 eine mittlere bis hohe Schwierigkeit auf. Die Trennschärfe lag für alle Items des CES und des COMOT-15 bei > 0,3 und war damit akzeptabel. Die Antworten erstreckten sich für alle Items auf die maximale Spannweite der einzelnen Items.

**Table 3:** 

Frage	MW	SD	Varianz	Trennschärfe	Itemschwierigkeit	Spannweite
*CES* (Abb. 1)						
a1	1,48	1,12	1,25	0,42	49,33	0–3
a2	3,09	1,76	3,08	0,49	64,8	0–5
a3	3,23	1,55	2,41	0,43	64,6	0–5
s1	1,75	1,19	1,42	0,47	35	0–5
s2	3,68	1,64	2,68	0,67	73,6	0–5
s3	3,84	1,35	1,83	0,53	76,8	0–5
s4	2,61	1,34	1,81	0,65	65,25	0–4
s5	2,06	1,39	1,93	0,47	41,2	0–5
s6	2,86	1,44	2,07	0,64	71,5	0–4
s7	4,01	1,44	2,06	0,68	80,2	0–5
m1	2,1	1,14	1,3	0,46	52,5	0–4
m2	3,43	0,89	0,79	0,5	85,75	0–4
m3	3,25	1,14	1,29	0,39	81,25	0–4
*COMOT-15* (Abb. 3)						
1	1,33	1,56	0,43	0,45	26,6	0–5
2	1,27	1,25	1,56	0,47	25,4	0–5
3	1,38	1,41	1,98	0,33	27,6	0–5
4	2,09	1,68	2,83	0,41	41,8	0–5
5	1,19	1,58	2,49	0,5	23,8	0–5
6	3,03	1,27	1,62	0,65	60,6	0–5
7	3,27	1,14	1,3	0,68	65,4	0–5
8	3,29	1,16	1,34	0,73	65,8	0–5
9	3,22	1,23	1,5	0,64	64,4	0–5
10	2,01	1,55	2,4	0,76	40,2	0–5
11	2,39	1,57	2,47	0,76	47,8	0–5
12	2,2	1,65	2,73	0,7	44	0–5
13	3,09	1,2	1,44	0,48	61,8	0–5
14	2,84	1,27	1,6	0,77	56,8	0–5
15	2,95	1,42	2,02	0,3	59	0–5

*CES *Chronic Ear Survey,* COMOT-15 *Chronic Otitis Media Test 15, *MW* Mittelwert, *SD* Standardabweichung

**Figure 4:**
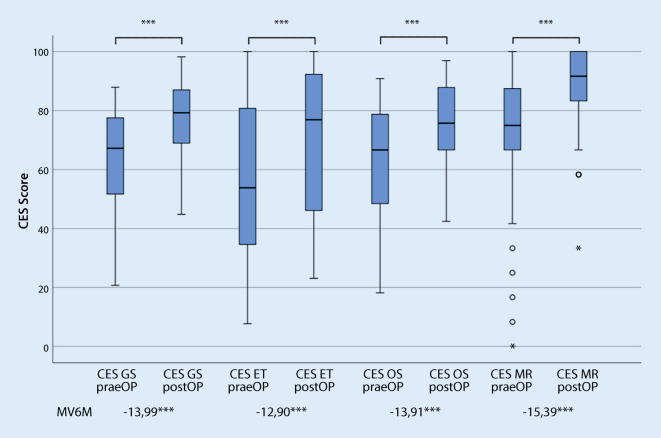

Im prä- und postoperativen Vergleich zeigte sich sowohl bei Nutzung des CES als auch des COMOT-15 eine signifikante Verbesserung der HRQoL im Gesamtscore und in allen Subscores (Abb. 4 und 5). Entsprechend dem Bewertungsvorschlag von Cohen konnte bei Nutzung des CES ein hoher, bei Anwendung des COMOT-15 ein mittlerer Effekt nachgewiesen werden. Betrachtet man die Subscores beider Messinstrumente, lässt sich für alle Subscores ein mittlerer Effekt darstellen (Tab. 2). Lediglich für den Subscore „Hörfunktion“ des COMOT-15 war die Responsivität gering.

**Figure 5:**
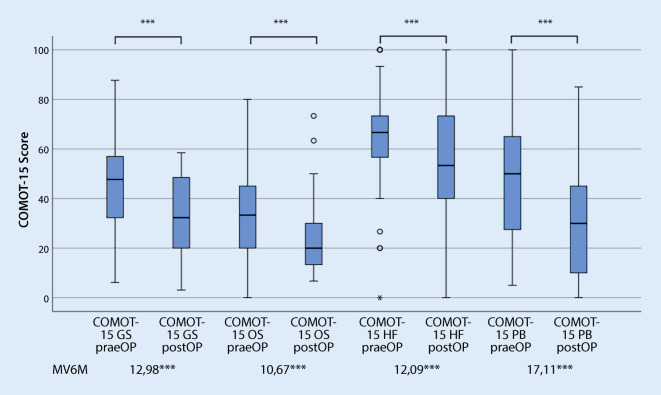

Für beide Messinstrumente zeigte sich insgesamt nur eine schwache Korrelation der postoperativen Gesamtscores mit der postoperativen Luftleitungsschwelle. Lediglich die Subscores „Symptome“ des CES und „Hörfunktion“ des COMOT-15 korrelierten moderat mit der Luftleitungsschwelle (Tab. 2).

Der CES und der COMOT-15 korrelieren stark im Gesamtscore (r = 0,68, 95 % CI 0,55–0,78). Der Subscore „Symptome“ beider Messinstrumente (r = 0,67, 95 % CI 0,53–0,77) sowie die Subscores „medizinische Ressourcennutzung“ (r = 0,69, 95 % CI 0,34–0,9) zeigten ebenfalls eine starke Korrelation.

Die lineare Regression zur Modellierung der Beziehung zwischen dem CES und dem COMOT-15-Gesamtscores ergab folgende Gleichung:

$$\text{Gesamtscore CES}=-0{,}56\times \text{Gesamtscore COMOT-15}+96{,}36$$

$$\text{Gesamtscore COMOT-15}=-0{,}68\times \text{Gesamtscore CES}+89{,}57$$

Zur Überprüfung eines möglichen Response-Shifts erfolgte der gepaarte t‑Test zwischen den jeweiligen Gesamtscores der präoperativen Befragung sowie der postoperativen Einschätzung der präoperativen Situation. Die Gesamtscores des CES und des COMOT-15 zeigten allesamt einen Shift hin zu einer besseren Lebensqualität (Tab. 2). Die Effektstärke (SES) zeigte beim CES (SES = −0,17) einen kleinen Effekt. Beim COMOT-15 (SES = 0,44) stellte sich hingegen ein mittlerer Effekt dar. In der Subscore-Analyse wurde für den Subscore „Hörfunktion“ des COMOT-15 mit einer SES von 0,59 ein relevanter positiver Response-Shift-Bias ermittelt. Der Subscore „psychisches Befinden“ (SES = 0,37) zeigte einen mittleren Effekt. Einzig der Unterpunkt „Ohrsymptomatik“ (SES = −0,2) des COMOT-15 wies einen negativen Response-Shift-Bias auf, der aber als kleiner Effekt eingestuft werden muss. Die Analyse des CES zeigte insgesamt kleine bis vernachlässigbare Effekte. Die negativen Werte in der Effektstärke sind auch hier als positiver Shift zu bewerten, da beim CES höhere Scores mit einer besseren Lebensqualität einhergehen, wohingegen bei dem COMOT-15 ein höherer Wert mit einer geringeren HRQoL gleichzusetzen ist.

## Diskussion

Unter der Annahme, dass die genutzten objektiven Parameter das Outcome einer chirurgischen Intervention nur unzureichend widerspiegeln, wird schon lange der zusätzliche Nachweis einer Verbesserung der HRQoL gefordert [10]. Für Patient:innen mit COM stehen im angloamerikanischen Raum eine Vielzahl von Messinstrumenten der HRQoL zur Verfügung, für welche es im deutschen Sprachraum kein validiertes Pendant gibt [12]. Hieraus resultiert eine fehlende Vergleichbarkeit internationaler Daten [17].

Die vorliegende deutsche Übersetzung des CES wies eine hohe interne Konsistenz auf. Die Cronbach-α-Werte lagen hier prä- und postoperativ (0,65–0,85) über den Werten der Validierung der englischen Version des CES (0,60–0,83 [25]). Bei der Bewertung der Test-Retest-Reliabilität ergaben sich für alle Subscores sowie den Gesamtscore Pearson-Korrelationskoeffizienten > 0,8, was für eine hohe Reliabilität der deutschen Version des CES spricht und sich mit den Daten der Originalpublikation deckt [25]. Darüber hinaus konnte nachgewiesen werden, dass die vorliegende Version des CES gut zwischen Patient:innen mit COM und Ohrgesunden unterscheiden kann. Auch zeigt sich eine gute Übereinstimmung mit der Gesamteinschätzung der Patient:innen in der Korrelation mit einer globalen gesundheitsspezifischen Frage.

Während die einzelnen Items des COMOT-15 auf Basis einer umfangreichen Einzelitemanalyse aus einer zunächst 31 Items umfassenden Erstversion des Messinstruments hervorgegangen ist [2], wurde im Rahmen der Validierung des CES keine Bewertung der einzelnen Items durchgeführt [16]. Die Selektion der Items des CES erfolgte auf Basis der inhaltlichen Einschätzung einer Expert:innengruppe. Trotz dieser möglichen methodischen Schwäche des CES wiesen die Einzelitems der deutschsprachigen Version des CES insgesamt auch zufriedenstellende Verteilungseigenschaften auf. Lediglich ein Item (Item m2, Erfassung der oralen Antibiotikagabe) war für die Patient:innen zu leicht, verfügte jedoch über eine gute Trennschärfe. Für den COMOT-15 konnten analog zur Validierungsstudie gute Verteilungsparameter für alle Items nachgewiesen werden.

Bei der Responsivität zeigte sich ein mittlerer Effekt für die Subscores und für den Gesamtscore des CES sogar ein großer Effekt, wohingegen sich bei Nutzung der englischsprachigen Originalversion nur moderate Effekte nachweisen ließen [25]. Im Vergleich zum COMOT-15 stellte sich für den Gesamtscore des CES eine höhere Änderungssensitivität heraus. Bei beiden Messinstrumenten war die höchste Responsivität für die Subscores „Ohrsymptome“ zu ermitteln. Der CES fokussiert aufgrund der Inhalte seiner Items insgesamt stark auf die Ausprägung von Ohrsymptomen, was an dieser Stelle zu seiner höheren Änderungssensitivität im Gesamtscore beiträgt. Der CES erwies sich dabei im Gesamtscore, aber auch im Subscore „Ohrsymptomatik“ im Vergleich zum COMOT-15 deutlich robuster gegenüber Response-Shift-Effekten. Diese Eigenschaften machen den deutschsprachigen CES insbesondere nützlich für Wiederholungsmessungen der HRQoL zur Erfassung von Therapieeffekten im Verlauf, wenn der Schwerpunkt auf der Ermittlung der subjektiven Beeinträchtigung durch die Ohrsymptomatik liegt.

Für den COMOT-15 zeigte sich ebenfalls eine hohe Änderungssensitivität. Jedoch fielen im Gegensatz zum CES deutlich ausgeprägtere Response-Shift-Effekte auf, welche für den Subscore „Hörfunktion“ sogar ein klinisch relevantes Ausmaß erreichten. Bislang existieren kaum Untersuchungen zur Bedeutung eines Response-Shifts für die Bewertung von HRQoL-Ergebnissen bei Patient:innen mit einer COM. Ein Response-Shift ist vordergründig dann zu erwarten, wenn sich Menschen mit kritischen und bedrohlichen Lebensereignissen auseinandersetzen [23]. Solche Ereignisse findet man v. a. in der Onkologie und bei chronischen Erkrankungen. Die Kenntnis des Einflusses eines Response-Shifts ist insbesondere für die Bewertung der Veränderungsmessung entscheidend. Je größer ein solcher Response-Shift zwischen 2 Messzeitpunkten ausfällt, desto geringer sind die Differenzwerte der Longitudinalmessung als wahre, quantitative Veränderung der HRQoL zu interpretieren. Andererseits müssen auch mögliche Verzerrungen durch fehlerhafte Erinnerungen berücksichtigt werden [19]. Für die anderen verfügbaren Messinstrumente zur Bestimmung der HRQoL bei COM wurde der Response-Shift bislang nicht bestimmt.

Der höchste Response-Shift zeichnete sich für den Subscore „Hörfunktion“ des COMOT-15 ab. Der COMOT-15 zielt im Vergleich zu den anderen derzeit verfügbaren HRQoL-Messinstrumenten am stärksten auf den Hörstatus und dessen Auswirkungen auf die HRQoL ab. Der Gesamtscore des Messinstruments, aber auch dessen Subscore „Hörfunktion“ zeigen trotzdem nur eine schwache Korrelation mit der ermittelten Luftleitungsschwelle. Aufgrund der Dominanz von hörbezogenen Items war eine stärkere Korrelation der Luftleitungshörkurve mit dem COMOT-15 im Vergleich mit dem CES zu erwarten. Jedoch unterschieden sich die ermittelten Korrelationskoeffizienten für die Gesamtscores beider Messinstrumente nicht. Bei der Betrachtung des CES fand sich der höchste Korrelationskoeffizient für den Subscore „Ohrsymptomatik“. Dieser Subscore wiederum korrelierte beim COMOT-15 nicht mit der Luftleitungshörschwelle. Diese Diskrepanz lässt sich dahingehend begründen, dass der Subscore „Ohrsymptomatik“ des CES im Gegensatz zum COMOT-15 den Hörverlust als Symptom miteinschließt. Als Konsequenz lässt sich für den Anwender ableiten, dass der COMOT-15 gegenüber dem CES für Untersuchungen bevorzugt werden sollte, in denen die subjektive Hörfunktion und deren Auswirkung auf die HRQoL analysiert werden soll. Bei Interventionsstudien und wiederholten Messzeitpunkten muss dann aber der hohe Response-Shift zur Interpretation der Ergebnisse berücksichtigt werden.

Die geringe Korrelation beider Messinstrumente mit der Hörfunktion legt an dieser Stelle nahe, dass auch andere patient:innenassoziierte Eigenschaften die krankheitsspezifische HRQoL beeinflussen. In einer klinischen Studie wurde für den COMOT-15 unlängst gezeigt, dass hier die Depressivität der Patient:innen den signifikanten Einflussfaktor darstellt, auch nach Adjustierung auf die anderen möglichen Einflussfaktoren Hörfunktion, Ausmaß der Pathologie und der individuellen Komorbiditäten [13]. Für den CES konnte eine Beeinflussung des Gesamtscores durch Gendereffekte, Diabetes mellitus, postoperative Komplikationen, den Bildungsstand und die operative Technik nachgewiesen werden [3].

Im CES wird im Gegensatz zum COMOT-15 die „medizinische Ressourcennutzung“ umfangreicher abgebildet. Während im COMOT-15 diesem Aspekt nur mit einem Einzelitem zur Erfassung der Häufigkeit der Arztbesuche Rechnung getragen wird, wird diese Domäne im CES anhand von 3 Items in einem eigenständigen Subscore erfasst. Trotz geringer Itemanzahl ergab sich für diesen Subscore in der Validierung der deutschsprachigen Version eine hohe interne Konsistenz. Es zeigte sich eine starke Korrelation des Ergebnisses des Einzelitems zur Ressourcennutzung des COMOT-15 mit dem Subscore des CES, sodass auch die Erfassung der medizinischen Ressourcennutzung im COMOT-15 mit nur einem Item als adäquat eingestuft werden kann.

Für den COMOT-15 zeigte sich eine stärkere Korrelation (r = 0,78) des Gesamtscore mit einer globalen Frage zur Gesamteinschätzung der HRQoL durch die Patient:innen im Vergleich zum CES (r = 0,51). Dass der CES stark auf die Ausprägung von Symptomen fokussiert, wird in diesem Messinstrument die HRQoL nur indirekt in den Subscores „Einschränkungen in Aktivitäten“ und „Ohrsymptome“ widergespiegelt. Hingegen enthält der COMOT-15 einen eigenständigen Subscore zur Erfassung des psychischen Befindens, welcher die stärkste Korrelation aller Subscores mit der globalen Frage aufwies (r = 0,73). Unter diesem Hintergrund ist eine Anwendung des COMOT-15 zu bevorzugen, wenn insbesondere die psychosoziale Dimension der HRQoL herausgearbeitet werden soll.

Die ähnlichen Inhalte des CES und des COMOT-15 resultieren insgesamt in einer signifikanten Korrelation der Gesamtscores und der übereinstimmenden Subscores „Symptome“ und „medizinische Ressourcennutzung“. Diese hohe konkordante Validität kann die Umrechnung beider Messinstrumente ermöglichen, um Daten zwischen verschiedenen Zentren oder Studien zu vergleichen. Diese modellierte Beziehung beider Messinstrumente sollte jedoch eine gemeinsame Anwendung beider Messinstrumente nicht ersetzen.

## Fazit für die Praxis

- Die deutschsprachige Version des Chronic Ear Survey (CES) weist zufriedenstellende psychometrische Kenndaten auf, sodass dessen Anwendung empfohlen werden kann.
- Aufgrund der hohen Änderungssensitivität des CES eignet er sich insbesondere für Interventionsstudien zur Bestimmung der prä- und postoperativen krankheitsspezifischen bzw. gesundheitsbezogenen Lebensqualität („health-related quality of life“, HRQoL).
- Der CES zeigt im Gegensatz zum Chronic Otitis Media Outcome Test 15 (COMOT-15) nur einen geringen Response-Shift, sodass er sich insbesondere für Untersuchungen mit mehreren Wiederholungsmessungen eignet.
- Aufgrund der hohen konkordanten Validität kann die Anwendung beider Messinstrumente (CES und COMOT-15) empfohlen werden.
